# *Helicobacter pylori* virulence factor CagA promotes tumorigenesis of gastric cancer *via* multiple signaling pathways

**DOI:** 10.1186/s12964-015-0111-0

**Published:** 2015-07-11

**Authors:** Xin Yong, Bo Tang, Bo-Sheng Li, Rui Xie, Chang-Jiang Hu, Gang Luo, Yong Qin, Hui Dong, Shi-Ming Yang

**Affiliations:** Department of Gastroenterology, Xinqiao Hospital, Third Military Medical University, Chongqing, 400037 P.R. China

**Keywords:** *Helicobacter pylori* CagA, Wnt/β-catenin, PI3K/Akt, p53

## Abstract

*Helicobacter pylori* (*H. pylori*) infection is strongly associated with the development of gastric diseases but also with several extragastric diseases. The clinical outcomes caused by *H. pylori* infection are considered to be associated with a complex combination of host susceptibility, environmental factors and bacterial isolates. Infections involving *H. pylori* strains that possess the virulence factor CagA have a worse clinical outcome than those involving CagA-negative strains. It is remarkable that CagA-positive *H. pylori* increase the risk for gastric cancer over the risk associated with *H. pylori* infection alone. CagA behaves as a bacterial oncoprotein playing a key role in *H. pylori*-induced gastric cancer. Activation of oncogenic signaling pathways and inactivation of tumor suppressor pathways are two crucial events in the development of gastric cancer. CagA shows the ability to affect the expression or function of vital protein in oncogenic or tumor suppressor signaling pathways via several molecular mechanisms, such as direct binding or interaction, phosphorylation of vital signaling proteins and methylation of tumor suppressor genes. As a result, CagA continuously dysregulates of these signaling pathways and promotes tumorigenesis. Recent research has enriched our understanding of how CagA effects on these signaling pathways. This review summarizes the results of the most relevant studies, discusses the complex molecular mechanism involved and attempts to delineate the entire signaling pathway.

## Introduction

*Helicobacter pylori* (*H. pylori*) is the most common human pathogen worldwide, infecting an estimated 50 % of the global population [1]. During *H. pylori* infection, sustained inflammation and abnormal epithelial proliferation may be major factors that lead to *H. pylori*-associated gastric diseases, such as gastritis, peptic ulcers, mucosa-associated lymphoid tissue lymphoma and gastric cancer. Further studies have indicated that *H. pylori* is responsible for several extragastric diseases, such as iron deficiency anemia and idiopathic thrombocytopenic purpura [1, 2]. Interesting associations have also been noted between H. pylori and other extragastric diseases, such as cardiovascular, neurological, hepatobiliary, colonic and pancreatic diseases [2]. However, gastric cancer is one of the most malignant types of tumor and represents a major health problem worldwide. The association of *H. pylori* with gastric cancer has received a great deal of attention and has been thoroughly studied. The World Health Organization and the International Agency for Research declared *H. pylori* to be a Group I human carcinogen for gastric cancer in 1994, and a prospective cohort study further implied that *H. pylori* is a necessary cause of gastric cancer [3]. *H. pylori* also affects the prognosis of gastric cancer. Fukase et al. performed a multi-center, open-label, randomized controlled trial to clarify that eradication of *H. pylori* should be performed after endoscopic resection of early gastric cancer to prevent the development of metachronous gastric cancer [4].

The majority of the *H. pylori*-infected population remains asymptomatic, and few individuals may develop gastric cancer. As the clinical outcomes caused by *H. pylori* infection are considered to be associated with a complex combination of host susceptibility, environmental factors and bacterial isolates [5]. The *H. pylori* genome shows genetic diversity among distinct isolates, and *H. pylori* pathogenicity is different in distinct isolates. Bacterial virulence factors exert an important influence in determining the clinical outcomes, and clinically isolated *H. pylori* strains are therefore classified according to bacterial virulence factors. The strongest candidates include the cag pathogenicity island (cag PAI) and vacuolating cytotoxin A (VacA). Clinically isolated *H. pylori* strains are often subdivided into two types according to the cag PAI-encoded cytotoxin-associated gene A (CagA) protein. Infections involving *H. pylori* strains that possess a functional cag PAI confer a higher risk for gastric cancer than those involving cag-negative strains [6]. The cag PAI is a 40 kb DNA fragment that encodes the CagA protein and functional components of a type IV secretion system (T4SS). The CagA protein, which is injected into gastric epithelial cells through the T4SS, behaves as a bacterial oncoprotein [7]. Ohnishi N et al. generated CagA transgenic mice that showed a significant increase in the incidence of gastric cancer. These results provide first direct evidence of the role of CagA as a bacterial oncoprotein that acts in mammals [8]. Meta-analyses further indicates that individuals infected with CagA-positive strains of *H. pylori* show an increased risk for gastric cancer over the risk associated with *H. pylori* infection alone [9, 10].

The molecular mechanism underlying CagA-positive *H. pylori*-induced gastric cancer has been widely studied. Translocation of CagA into gastric epithelial cells is the first step in the processes of CagA-induced tumorigenesis. Several different Cag proteins are involved in the translocation of CagA. One of these proteins, CagL, functions as a component of the T4SS that binds to and activates α5β1 integrin receptors, triggering the delivery of the bacterial effector protein CagA to the cytoplasm of host cells [11]. Additional Cag proteins (CagY, CagI, CagA) have also been shown to bind β1 integrin and permit translocation of the bacterial effector protein CagA [12, 13]. Another structural component, CagE, was reported to be essential for CagA translocation. Furthermore, infection with *H. pylori* can induce phosphatidylserine (PS) externalization in epithelial cells, and CagA then interacts with the externalized PS to initiate its entry into cells [14]. Collectively, these findings indicate that *H. pylori* exploits host cell surface molecules such as integrins and PS to deliver CagA to the host cells. Additionally, CagA translocation requires energy-dependent host cell processes distinct from endocytic pathways. Cytomembrane cholesterol and actin polymerization are also necessary for CagA translocation [14].

Once the protein has entered these target cells, parts of CagA molecules are tyrosine-phosphorylated by Src and Abl family kinases within several repeat Glu-Pro-Ile-Tyr-Ala (EPIYA) motifs, while other CagA molecules remain unphosphorylated [15–17]. CagA then binds to various signaling proteins and causes dysregulation of multiple signaling pathways in either a phosphorylation-dependent or phosphorylation-independent manner [18]. Phosphorylated CagA causes epithelial cells elongation and scattering, a morphology was originally referred to as the “hummingbird phenotype”, due to its effect on host cell signaling pathways, such as the extracellular signal-regulated kinase (ERK)/mitogen-activated protein kinase (MAPK) pathway, by interacting with SHP2, Grb2 and Crk/Crk-L [19, 20]. On the other hand, unphosphorylated CagA interacts with various signaling proteins, such as Met, E-cadherin, Grb2 and Par1b, and then activates corresponding signaling pathways, such as the phosphatidylinositol 3-kinase/Akt (PI3K/Akt) signaling pathway, nuclear factor-κB (NF-κB) signaling pathway, Wnt/β-catenin signaling pathway and Ras signaling pathway, among others [21–23]. These interactions and the activation of these signaling pathways contribute to the epithelial proliferation and pro-inflammatory processes as well as the disruption of cell-to-cell junctions, or loss of cell polarity.

This review focuses on recent findings regarding CagA, related to various kinds of vital signaling proteins and several classic signaling pathways. In combination with previous studies on corresponding proteins or signaling pathways, we hope to discuss the possible molecular mechanism underlying the CagA-induced abnormal expression of vital signaling proteins and the dysregulation of signaling pathways, and to further recognize the relationship of CagA and the clinical outcomes of *H. pylori* infection, especially gastric cancer, which is the most severe outcome.

### CagA mediates dysregulation of the Wnt/β-catenin signaling pathway

The Wnt/β-catenin signaling pathway is a key pathway regulating embryonic development and adult tissue homeostasis. Aberrant Wnt/β-catenin signaling plays an essential role in disease pathogenesis, especially in tumorigenesis and progression [24, 25]. The Wnt/β-catenin signaling pathway is highly conserved among species, and the core of this pathway is the versatile protein β-catenin, encoded by CTNNB1. The canonical Wnt/β-catenin signaling pathway is activated by a secreted extracellular Wnt ligand that binds to frizzled (Fzd) receptors and their co-receptors the low-density lipoprotein receptor related protein (LRP) families. The activated Fzd receptor switches on the intracellular signaling cascade. Then the phosphorylation of LRP activates the disheveled protein (Dvl), which in turn inactivates a complex of proteins collectively termed the “destruction complex”, including the core proteins glycogen synthase kinase-3β (GSK-3β), Axin, adenomatosis polyposis coli (APC) and casein kinase-1 (CK-1). In the absence of Wnt ligands or the presence of abundant competitive ligands, such as Wnt inhibitory factor-1(WIF-1), secreted Frizzled related proteins (sFRPs), or Dickkopf family proteins (DKKs), the destruction complex phosphorylates the N-terminus of cytosolic β-catenin, and targets it for proteasomal degradation, thereby maintaining β-catenin at low baseline cytosolic levels. However, in the presence of Wnt ligands, cytosolic β-catenin can not be phosphorylated due to inactivation of the destruction complex. As a result, β-catenin is stabilized and accumulates in the cytoplasm and subsequently translocates to the nucleus. In the nucleus, β-catenin interacts with the T cell factor/lymphoid enhancer factor (TCF/LEF) family of transcription factors to induce target gene transcription [26, 27].

Dysregulation of the Wnt/β-catenin signaling pathway is widely implicated in gastrointestinal cancers, including colorectal cancer and gastric cancer [27, 28]. Mutation of pathways components (eg. Axin and β-catenin), inhibition of antagonists (eg. sFRPs) or crosstalk with other signaling pathways leads to continuous dysregulation of the Wnt/β-catenin signaling pathway. Infection by Pathogenic microorganisms, such as *H. pylori*, Hepatitis virus, is another important factor influencing the Wnt/β-catenin signaling pathway [29–33]. Franco et al. reported that CagA-positive *H. pylori* altered β-catenin localization and increased β-catenin nuclear accumulation in gastric epithelial cells AGS. Along with β-catenin nuclear translocation, the Wnt/β-catenin signaling pathway is activated. These authors also observed this phenomenon in gerbil gastric mucosae and human gastric mucosae colonized by CagA-positive *H. pylori* [29]. Another study further confirmed that the nuclear translocation of β-catenin and subsequent activation of the Wnt/β-catenin signaling by CagA required the EPIYA repeat region and was independent of CagA tyrosine phosphorylation [30]. Neal et al. generated transgenic zebrafish expressing CagA and found the Wnt/β-catenin target genes significant upregulation in the transgenic zebrafish. The functional consequences of the CagA-activated Wnt/β-catenin signaling pathway included increased intestinal proliferation. These results provided in vivo evidence of CagA-induced Wnt/β-catenin signaling pathway activation [31].

**Fig. 1:**
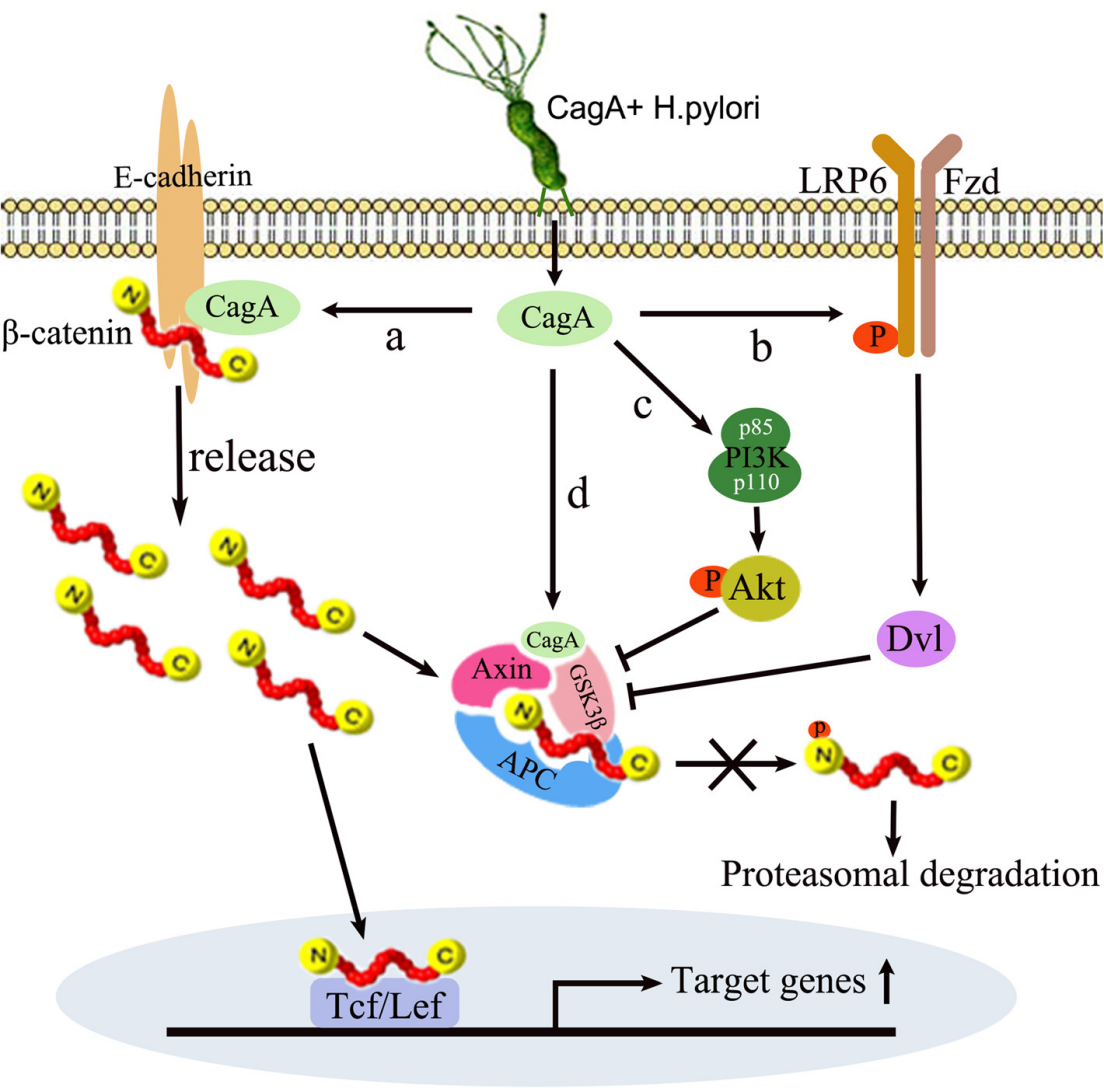
CagA mediates dysregulation of the Wnt/β-catenin signaling pathway. a. CagA competitively combines with E-cadherin and disrupts the E-cadherin/β-catenin complex formation, causing cytoplasmic and nuclear accumulation of β-catenin. b. *H. pylori* induces rapid phosphorylation and activation of LRP6. c. CagA induces GSK-3β inactivation via the PI3K/Akt signaling pathway. d. CagA binds GSK-3β directly and depletes GSK-3β activity, inhibiting the phosphorylation and proteasomal degradation of cytosolic β-catenin

Under physiological conditions, β-catenin interacts with the cytoplasmic tail of E-cadherin to form adherens junctions between epithelial cells. However, upon infection with CagA-positive *H. pylori* strains, CagA will become competitive in combination with E-cadherin and disrupt complex formation between E-cadherin and β-catenin, causing cytoplasmic and nuclear accumulation of β-catenin. In addition, the interaction between CagA and E-cadherin is independent of CagA tyrosine phosphorylation, but the specific binding sites involved are not clear (Fig. 1a) [22]. Therefore, they may be directly bound or indirectly bound via another component of the adherens junction complex. Although the details of the interaction between CagA and E-cadherin remain to be elucidated, this research provided the molecular mechanism of CagA deregulation of β-catenin and subsequent activation of the canonical Wnt/β-catenin signaling pathway. AGS gastric epithelial cells are commonly used in the study of *H. pylori* because these cells are highly susceptive to *H. pylori* infection. Interestingly, AGS do not form E-cadherin/β-catenin complexes due to a lack of E-cadherin expression, and CagA is therefore not able to release of β-catenin from the E-cadherin/β-catenin compelx. Nevertheless, CagA still increases the cytoplasmic and nuclear accumulation of β-catenin in AGS cells and activates the Wnt/β-catenin signaling pathway [21, 29]. Thus, there may be other mechanisms through which CagA regulates β-catenin and the Wnt/β-catenin signaling pathway.

Wnt ligands/receptors or the components of the destruction complex may be involved in the CagA-positive *H. pylori*-induced activation of the Wnt/β-catenin signaling pathway. It has been reported *H. pylori* induces rapid activation of the Wnt/β-catenin signaling pathway co-receptor LRP6, which is dependent on proteins of Dvl family (Fig. 1b) [34]. Although this process was found to be independent of CagA, *H. pylori* lacking a functional T4SS failed to induce LRP6 phosphorylation and activation. It is still unclear how *H. pylori* activates LRP6 and whether there is a direct relationship between *H. pylori* and Dvl. Sokolova et al. reported that *H. pylori* suppressed GSK-3β activity to promote β-catenin activity in a CagA-independent manner [35]. Subsequently, Nakayama et al. reported that *H. pylori* VacA induced the phosphorylation and inactivation of GSK-3β through the PI3K/Akt signaling pathway, and subsequent nuclear translocation of β-catenin regulated transcriptional activity [36]. Tabassam et al. came to a similar conclusion, that *H. pylori* induced GSK-3β inactivation via the PI3K/Akt signaling pathway, but their results showed that CagA was responsible for GSK-3β inactivation and downstream β-catenin activation (Fig. 1c) [37]. These opposing results may be due to the differences in the cell lines and *H. pylori* strains used in the research. Sokolova et al. mainly employed MDCK cells and an *H. pylori* strain P1 expressing CagA. They confirmed that CagA could be translocated and phosphorylated at tyrosine residues in both AGS and MDCK cells, but they did not verify the following results in AGS cells. Similarly, Nakayama et al. only used the AZ-521 cell line in their research, and this cell line has been used relatively less frequently in research on *H. pylori*. Recently, Korean researchers explained *H. pylori* CagA-induced GSK-3β inactivation from another perspective. They indicated that CagA could directly bind GSK-3β and deplete GSK-3β activity. Furthermore, they found that the C-terminal region of CagA harbored an EPIYA motif and a multimerization domain that played an important role in the binding and depletion of GSK-3β (Fig. 1d) [38]. This result is consistent with previous findings showing that the EPIYA repeat region is responsible for β-catenin activation [30]. Taken together, these results show that CagA mediates dysregulation of the Wnt/β-catenin signaling pathway via effecting the release and degradation of β-catenin.

### CagA activates PI3K/Akt and the downstream signaling pathways

PI3K, which is involved in tumorigenesis, is a heterodimer consisting of a p85 regulatory subunit and a p110 catalytic subunit. The main function of PI3K is to phosphorylate phosphatidylinositol in the cytomembrane and convert it into phosphatidylinositol 3, 4, 5-triphosphate (PIP3). PIP3 interacts with the pleckstrin homology (PH) domain of a ser/thr kinase Akt and allows Akt phosphorylation at Ser473 or Thr308. This phosphorylation activates Akt, which in turn, activates or inactivates downstream tyrosine kinase- and G-protein-coupled receptors in turn. GSK-3β, NF-κB and mammalian target of rapamycin (mTOR) are key representative of PI3K/Akt downstream target proteins [37, 39–44], and the MEK/ERK signaling pathway is a representative of PI3K/Akt downstream signaling pathway [45].

The PI3K/Akt signaling pathway is hyperactive in some cancers, including gastric cancer [46, 47]. *H. pylori* infection is a major factor in the activation of PI3K/Akt and downstream signaling pathways [21, 37, 44]. The activation of PI3K/Akt signaling pathways is a response to growth factors (eg. epidermal growth factor, hepatocyte growth factor). As the specific receptors of epidermal growth factor and hepatocyte growth factor, the epidermal growth factor receptor (EGFR) and c-met, respectively, mediate the activation of the PI3K/Akt signaling pathway. *H. pylori* infection induces the phosphorylation of EGFR Tyr 992 and the transactivation of EGFR, which is dependent on CagA and another virulence factor, OipA. As a result, the PI3K p85 subunit is activated, and two specific phosphorylation sites (Thr 308 and Ser 473 of Akt) are phosphorylated. Importantly, the CagA mutation reduces the activation of Akt Thr 308, whereas the oipA mutation reduces the levels of Akt Ser 473 in response to *H. pylori* infection. Double CagA/OipA mutation results in complete inhibition of the phosphorylation of Akt Ser 473, confirming that CagA mainly affects the phosphorylation of Akt Ser 473 [37]. Interestingly, EGFR is activated by *H. pylori* during early infection, but *H. pylori* CagA inactivates EGFR during prolonged infection via reducing the phosphorylation of EGFR tyrosine residues [48]. This research revealed that CagA-positive *H. pylori* regulates EGFR activation and inactivation to support persistent infection. But it is regrettable that the EGFR/PI3K/Akt signaling pathway is not mentioned in this paper. Thus, the role of EGFR in *H. pylori*-induced signaling pathways is not completely clear and needs to be investigated more thoroughly in the future. However, it has been confirmed that c-met is activated in *H. pylori*-infected conditions, and CagA is able to interact with activated c-met [21, 49] Moreover, the C-terminal region of CagA, which is designated CRPIA (conserved repeat responsible for phosphorylation-independent activity), is involved in the interaction with c-met and the activation of the downstream PI3K/Akt signaling pathway. As a results of CagA-induced PI3K/Akt signaling pathway activation, GSK3β, which is a downstream target of PI3K/Akt, is inactivated, and β-catenin transcription is subsequently activated [21, 37]. In other words, there is a crosstalk between the CagA-induced Wnt/β-catenin signaling pathway and the PI3K/Akt signaling pathway.

Recently, Zhang et al. found CagA EPIYA repeat region was also involved in the activation of PI3K/Akt signaling pathway [50]. The EPIYA repeat region of CagA is classified into 4 tyrosine phosphorylation motifs (TPMs), A-, B-, C- or D-TPMs. Western CagA has A-, B-, and C-TPMs, while East Asian CagA possesses A-, B-, and D-TPMs [51, 52]. Zhang et al. demonstrated CagA activated PI3K/Akt signaling pathway by interaction with PI3K via a functional B-TPM. Through database searching and silico analysis, they revealed a strong non-random distribution of the B-TPM polymorphisms (A/T polymorphism, including EPIYA and EPIYT) in Western *H. pylori* isolates. And Matsunari et al. previously also reported that B-TPM polymorphisms (EPIYT, EPIYA, ESIYA and so on) and that EPIYT of B-TPM was more predominant in Western *H. pylori* isolates and EPIYA of B-TPM was more predominant in East Asian *H. pylori* isolates [53]. Interestingly, during co-culture with AGS cells, an *H. pylori* strain with EPIYT B-TPM had higher affinity to PI3K and significantly enhanced induction of PI3K/Akt signaling pathway, compared to the isogenic strain with EPIYA B-TPM [50]. These results suggest that the A/T polymorphisms in B-TMP could regulate PI3K/Akt signaling pathway activity through influencing the interaction between CagA and PI3K. In addition, *H. pylori* strain with EPIYT B-TPM induced less hummingbird cells and IL-8 levels than the isogenic strain with EPIYA B-TPM [50]. In this regard, the differential EPIYT and EPIYA functions might be part of the reason that the incidence of gastric carcinoma is much higher in East Asian countries than in Western countries. However, it is regrettable that there is no direct result confirms the relationship between PI3K/Akt signaling and hummingbird phenotype and IL-8 levels. Indeed, it is difficult to use a simple linear relationship to clarify the regulation of PI3K/Akt signaling on hummingbird phenotype and IL-8 levels, because (i) PI3K/Akt downstream target proteins or signaling pathways are pleiotropic and have complex crosstalk with other signaling pathways, (ii) *H. pylori* could induce AGS hummingbird phenotype and IL-8 production through multiple CagA-mediated mechanisms.

NF-κB, which is a downstream target of PI3K/Akt, is a crucial regulator of many cellular processes, including inflammation, immune responses and tumorigenesis. NF-κB is a p65/p50 heterodimer that forms a complex with cytoplasmic inhibitors (eg. IκB) in resting condition. PI3K/Akt can activate IκB kinase (IKK), which phosphorylates IκB and subsequently releases NF-κB from the complex. Then NF-κB translocates to the nucleus, and phosphorylation of the p65 subunit plays a key role in the NF-κB-mediated transcriptional response. It has been accepted that *H. pylori* cag PAI is required for NF-κB activation [54, 55], but the role of CagA in the regulation of NF-κB activity is still a subject of intense discussion. Early studies found that *H. pylori* induced NF-κB activation in time-, multiplicity of infection-, and cag PAI-dependent manners [54]. Recently, Sokolova further confirmed that *H. pylori* cag PAI encoding T4SS was required for activation of NF-κB and release of downstream IL-8, but CagA had only a partial or minor role in NF-κB activation and IL-8 release at early infection [55]. In contrast, Brandt et al. found that CagA could activate NF-κB and induce downstream IL-8 release in a time-dependent manner [56]. In this study, CagA activates NF-κB and induces IL-8 induction via the MEK/ERK signaling pathway. Subsequently, Kim et al. constructed CagA and its fragments eukaryotic expression vectors, and further confirmed that NF-κB activation and IL-8 release induced by CagA occurred via the MEK/ERK signaling pathway activation [57]. Recently, Kang et al. compared different CagA-positive and -negative *H. pylori* strains for their ability to activation of NF-κB in different gastric cancer cells. They confirmed that CagA was required for *H. pylori*-induced activation of NF-κB and that CagA selectively induced Phospholipase D1 expression via NF-κB [58]. The different *H. pylori* strains used by different laboratories may be responsible for different components of *H. pylori* that drive NF-κB signaling pathway. However, the time point of *H. pylori* infection may be the main factor determing which component drive NF-κB signaling pathway. Most of the previous studies compared different CagA-positive and -negative *H. pylori* strains for their ability to activation of NF-κB at early time points of infection (less than 3 h). They found *H. pylori* induced NF-κB activation and IL-8 release in a CagA-independent manner [54, 55]. Interestingly, during persistent infection (more than 12 h), NF-κB activation and IL-8 release further increased in a CagA-dependent manner [56, 58]. It is may be meaningful to explore how T4SS initiate NF-κB signaling pathway and how CagA is involved in NF-κB activation during persistent infection. Transforming growth factor-β-activated kinase 1 (TAK1) is a key regulator of signal transduction cascades that lead to the activation of IKK and NF-κB. CagA physically interacts with TAK1 and enhances the activity of TAK1, which is required for NF-κB activation by CagA [59]. However, a later study has shown that this is a misinterpretation because the antibody against TAK1 immunoprecipitated to some extent the CagA protein by unknown reasons, while in a reverse immunoprecipitation using a CagA antibody they also could not recognise co-immunoprecipitated TAK1 [60]. Although they indicate TAK1 is not a target of the *H. pylori* CagA, TAK1 is required for *H. pylori*-induced NF-κB activation in a T4SS-dependent and CagA-independent manner during early infection. Actually, *H. pylori*-induced interaction between TAK1 and IKK complex may be involved in NF-κB initial activation in a T4SS-dependent manner during early infection, while CagA may be play a role in extend the activation of NF-κB during persistent infection because CagA takes more time to be delivered into the host cells and regulates its target proteins or signaling pathways. As mentioned in the previous studies, CagA need more time to enhance NF-κB activity and increase IL-8 release during persistent infection [56, 58]. Therefore, further studies are necessary to elucidate the mechanisms of *H. pylori*-induced interaction between TAK1 and IKK complex during early infection and the exact function of CagA in the regulation of NF-κB activity during persistent infection.

*H. pylori* CagA has the ability to activate of the PI3K/Akt signaling pathway, but the regulation of the PI3K/Akt downstream signaling pathways by *H. pylori* is still not completely clear. As an upstream regulator of NF-κB, PI3K/Akt is not mentioned in *H. pylori*-mediated activation of NF-κB. Similarly, it has been reported that *H. pylori* activates the PI3K/Akt/mTOR signaling pathway in a CagA-independent manner [44]. As we mentioned above, CagA and OipA can phosphorylate Akt and activate the PI3K/Akt signaling pathway, but only OipA is required for *H. pylori*-induced inactivation of the Forkhead transcription factors of class O (FoxO) family members FoxO1 and FoxO3a, which are the downstream of PI3K/Akt [61]. These findings indicate that *H. pylori* can activate PI3K/Akt and downstream signaling pathways via different molecules and mechanisms, maintaining the dysregulation of these signaling pathways and forming a complex network.

### The role of CagA in other oncogenic signaling pathways

Similar to the Wnt/β-catenin signaling pathway, the Hedgehog (Hh) signaling pathway plays a critical role in embryonic development, adult tissue homeostasis and tumorigenesis [62]. *H. pylori* infection induces upregulation of sonic hedgehog (Shh), which activates the Hh signaling pathway in gastric cancer cells. Moreover, Shh overexpression is CagA-dependent and is mediated through the NF-κB signaling pathway [63]. Shh is mainly expressed in parietal cells, influencing fundic gland differentiation and function. Recently, Schumacher et al. used a mouse model that expresses Shh fused to green fluorescent protein, in place of wildtype Shh to visualize Shh ligand expression in response to *H. pylori* infection in vivo. They found that *H. pylori* induced Shh overexpression in parietal cells that was consistent with the expression pattern observed in the native tissue. Furthermore, they confirmed that NF-κB signaling mediated *H. pylori*-induced Shh overexpression and that CagA is involved in this process [64]. In addition, in the early stages of H pylori infection, *H. pylori*-induced Shh overexpression from parietal cells acts as a macrophage chemoattractant to drive the innate immune response during the initiation of gastritis [65].

Since it was first discovered, the c-Jun NH2-terminal kinase (JNK) signaling pathway has been demonstrated to exhibit both tumor suppressor and pro-tumorigenic functions in different cell types and organs [66, 67]. Infection with *H. pylori* has been shown to activate the JNK signaling pathway, and CagA is an important mediator of the activation of this signaling pathway during infection [68, 69]. Wandler et al. used transgenic Drosophila to express CagA, and they found that CagA triggered JNK signaling pathway activation, which caused apoptosis in epithelial cells. Interestingly, when these authors employed a Ras oncogene-overexpression Drosophila metastasis model, they found that coexpression of CagA could enhance the growth and invasive potential of tumor cells through activation of the JNK signaling pathway [69]. This finding indicates that, in addition to the presence of virulence factors (eg. CagA), host genetics must also play an important role in determining the outcome of *H. pylori* infection. Indeed, *H. pylori* infection can persist for many years before the occurrence of gastric cancer. Therefore, JNK-mediated apoptosis may be an effective mechanism for limiting pathogenic effects and protecting the gastric epithelium in early infection. Under persistent *H. pylori* infection and the influence of other factors, accumulation of genetic mutations is observed. Following the acquisition of an oncogenic mutation, CagA-mediated JNK signaling pathway activation promotes tumor progression. It will be particularly valuable to further confirm the role of CagA-mediated JNK signaling pathway activation in clinical tissue samples, but there has been no report of such studies to date.

Dysregulation of the Janus kinase (JAK)/signal transducers and activators of transcription 3 (STAT3) signaling pathway is observed in many cancers including gastric cancers, and it correlates with both tumor progression and a poor prognosis. Jackson and colleagues found that STAT3 activation was more pronounced in CagA-positive *H. pylori*-infected gastric tissue [70]. In this study, as the majority of bacterial pathogens mediate STAT3 activation via autocrine IL-6, it was found that IL-6 expression was increased after *H. pylori* infection, and both IL-6 and IL-11 were strongly up-regulated in gastric cancer tissue. In epithelial cells infected with *H. pylori*, STAT3 tyrosine phosphorylation, nuclear translocation and transcriptional activity are dependent on unphosphorylated CagA. Although *H. pylori* CagA-mediated STAT3 activation requires IL-6 and the gp130 receptor, autocrine activation of STAT3 by IL-6 and IL-11 is not involved in *H. pylori* CagA-mediated STAT3 activation [71]. Further studies may be required to elucidate the exact mechanism underlying the interaction between CagA and these relevant receptors. Moreover, *H. pylori*-mediated STAT3 activation shows the ability to manipulate host immunity and facilitate immune evasion [72, 73]. Recent evidence indicates that CagA increases the expression of Gram-positive specific bactericidal lectin, regenerating islet-derived (REG) 3γ, in gastric epithelial cells via activation of the STAT3 signaling pathway [72]. While the functional basis of this response is not entirely clear, these findings indicate that CagA-medicated REG3γ overexpression may abolish the fitness of co-habiting Gram-positive bacteria and reduce the competition for resources between *H. pylori* and Gram-positive bacteria in the gastric mucosal niche. Finally, *H. pylori* may rebuild the gastric microbiome and manipulate host immunity to favor its own survival. The ability to evade the host immune response is another crucial factor in the survival of *H. pylori* in the host gastric mucosae. As DCs are key modulators of the host adaptive immune response, they are ideal targets for the pathogen’s immunity-manipulating efforts. Several studies indicate that *H. pylori* infection promotes the development of tolerogenic dendritic cells (DCs) in a coculture system and in murine models [74, 75]. Recently, Rizzuti and colleagues found that *H. pylori* activated the STAT3 signaling pathway in bone marrow-derived DCs (BMDCs). Then BMDCs secrete IL-10, which activates STAT3 in DCs, thereby blunting DC maturation, inducing the tolerogenic DCs [73]. This study describes another novel mechanism of *H. pylori* facilitation of immune evasion to maintain its persistence.

The regulation of signaling pathways may be influenced by the CagA tyrosine phosphorylation status. It has been reported that unphosphorylated CagA showed preferentially activates of JAK/STAT3, whereas phosphorylated CagA enhances SHP2 binding activity and ERK/MAPK signaling pathway activation [76]. These findings indicate that the CagA tyrosine phosphorylation status affects the signal switch, providing a novel mechanism explaining *H. pylori*-mediated signaling pathways. Unphosphorylated and phosphorylated CagA often exist at the same time therefore, STAT3 and ERK/MAPK signaling activation must be abnormal during *H. pylori* infection. In support of this hypothesis, a research group observed significantly increased STAT3 and ERK/MAPK signaling activation in *H. pylori*-infected gastric tissue, which was further enhanced in the presence of CagA-positive *H. pylori* strains [70]. Recent evidence indicates that IL-22 promotes gastric cancer development via activation of the STAT3 and ERK signaling pathways [77]. In this study, while gastric cancer cells were co-cultured with IL-22-expressing cancer-associated fibroblasts (CAFs) from human gastric cancer tissues, the invasive ability of the gastric cancer cells was significantly enhanced through activation of the STAT3 and ERK signaling pathways. In fact, *H. pylori* infection also stimulates peripheral mononuclear cells and CD4-positive T cells to secrete IL-22, and IL-22 subsequently induces the expression of antimicrobial proteins (eg. RegIIIα and lipocalin-2) in gastric epithelial cells [78]. In this regard, IL-22 may play a protective role in gastric mucosae infected by *H. pylori*, but more studies suggest that IL-22 may lead to pathological inflammation and thereby promote tumorigenesis and progression via STAT3 activation [77–80]. Th1 and Th17 are major T cell subsets that produce IL-22, and *H. pylori* is ability to induce Th1 and Th17 responses [81, 82]. Therefore, it is necessary to confirm that (i) the role of *H. pylori*-mediated upregulation of IL-22 in gastric cancer cells, (ii) the activation of STAT3 dependent on *H. pylori*-mediated IL-22 and/or *H. pylori* CagA, (iii) the role of CagA in *H. pylori*-mediated upregulation of IL-22.

In addition, H. pylori-mediated signaling (including p21-activated kinase1 and ERK1/2) to actin-binding protein cortactin could regulate cell scattering and elongation in a T4SS dependent manner [83, 84]. Injected CagA further involves in the interaction of cortactin with downstream focal adhesion kinase (FAK), and this interaction increases FAK activity, which is important for cell scattering and elongation phenotype [83]. Cortactin plays a central role in host signaling and involves in a variety of cellular processes, including tumorigenesis, invasion and metastasis. Thus, further studies may be required to elucidate the role of *H. pylori-*hijacked cortactin in tumorigenesis in vivo. Furthermore, CagA-positive *H. pylori*-induced activation of the Src/MEK/ERK signaling pathway is involved in the upregulation of α-enolase and ornithine decarboxylase, implying the progression of gastric diseases [85, 86]. What’s more, *H. pylori* is involved in protein kinase C (PKC) signaling pathway activation through PI3K, phospholipase Cγ and Ca^2+^. As a result, PKC contributes to c-Fos upregulation and activator protein-1 activation, leading to overexpression of matrix metalloproteinase-1 [87]. Due to the large number of the related studies, it is difficult to list all of *H. pylori*-mediated oncogenic signaling pathways. In other words, there are several known mechanisms underlying *H. pylori*-mediated gastric cancers.

### The role of CagA in tumor suppressor pathways

**Fig. 2:**
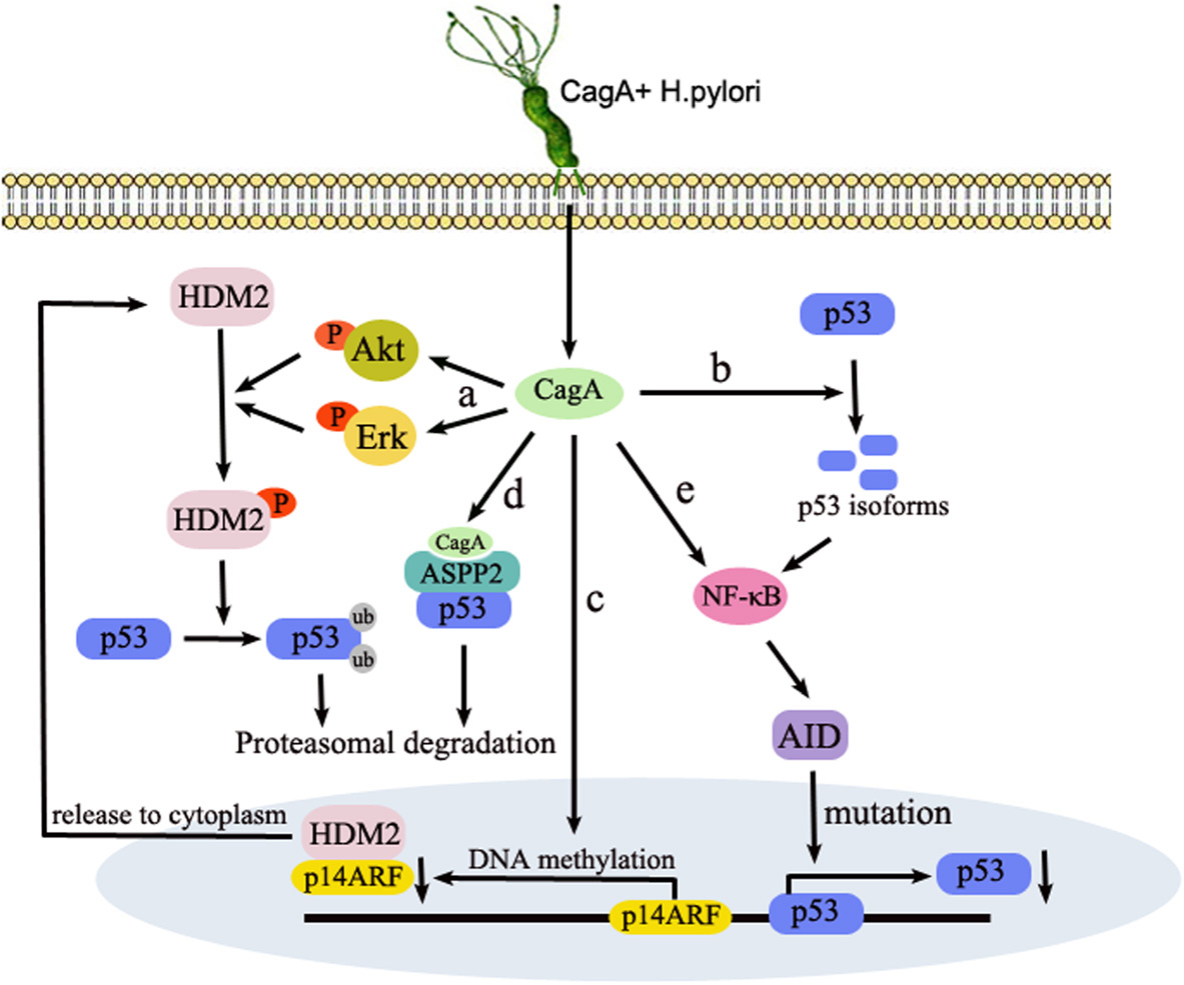
The role of CagA in p53 regulation. a. CagA phosphorylation and activation of HDM2 is mediated by Akt or ERK activation. b. CagA plays a crucial role in p53 shifting to inhibitory p53 isoforms. c. CagA-induced hypermethylation of the p14ARF promoter results in a decrease in p14ARF protein levels that is not sufficient to sequester HDM2 in the nucleus. d. CagA interacts with ASPP2 to recruit and bind p53, which is then degraded by the proteasome. e. CagA induces aberrant expression of AID via NF-κB and thereby elicits a high mutation frequency in p53

Tumorigenesis is considered to occur as a multifactorial events. The activation of oncogenes and the inactivation of tumor suppressor genes are two key events in most cancers. Aberrant activation of oncogenes can be counteracted by tumor suppressor genes. It is more significant to enrich and explain the tumorigenic mechanism of *H. pylori*. In addition to activating several oncogenic signaling pathways, *H. pylori* also plays a key role in the inactivation of tumor suppressor pathways. p53 is a key tumor suppressor, and inactivation of p53 is a critical step in tumorigenesis and progression. Previous reports showed that *H. pylori* infection increased p53 levels in the gastric mucosae [88–90]. Subsequently, Wei et al. observed that p53 levels were dynamically altered in *H. pylori*-infected mongolian gerbil gastric tissues and cell lines. Consistent with previous reports, these authors found that p53 was increased following *H. pylori* infection for 4–6 h in mongolian gerbils, however, p53 decreased rapidly following the initial increase. Interestingly, p53 was shown to be increased again under continuous *H. pylori* infection for 12 weeks [91]. In this study, the authors confirmed that CagA-positive *H. pylori* phosphorylated Human Double Minute 2 (HDM2, a main E3 ubiquitin ligase), which induced p53 degradation. In addition, *H. pylori*-induced phosphorylation and activation of HDM2 could be mediated by Akt or ERK activation (Fig. 2a) [91, 92]. Cellular stresses, such as *H. pylori*-induced activation of oncogenic signaling pathways, may be responsible for the initial increase of p53. The second increase of p53 may be driven by DNA damage known to be associated with inflammatory processes. In another report, Wei et al. indicated that *H. pylori* could induce upregulation of truncated p53 isoforms that inhibit p53 function and increase the transcriptional activity of NF-κB in gastric epithelial cells (Fig. 2b) [93]. Taken together, these findings indicate that DNA damage-mediate upregulation of p53 may be shifted to inhibitory p53 isoforms by CagA-positive *H. pylori*. Inhibition of p53 may allow *H. pylori* to alter the cellular homeostasis, without apoptosis or triggering cell cycle arrest.

The p14ARF is a tumor suppressor that inhibits the proteasomal degradation of p53 by sequestering HDM2 and inhibiting its E3 ligase activity [94]. *H. pylori* CagA-induced hypermethylation of the p14ARF promoter results in a decrease of p14ARF protein levels that is not sufficient to inhibit HDM2 and ARF-BP1 (another E3 ubiquitin ligase) activity, and then HDM2 and ARF-BP1 then facilitates the degradation of p53 (Fig. 2c) [95]. This study provides a novel mechanism in which the CagA-mediated degradation of p53 is controlled by two E3 ubiquitin ligases that are activated due to p14ARF promoter hypermethylation and downregulation of p14ARF protein levels. Apoptosis-stimulating protein of p53 (ASPP2) is best known for its role as a p53-binding protein and has also shown to act as a tumor suppressor. Following *H. pylori* infection, CagA shows the ability to interact with ASPP2 to form a complex. After this interaction, ASPP2 recruits and binds p53, which is then degraded by the proteasome. Although the formation of a ternary complex between CagA, ASPP2, and p53 has not been detected, it has been confirmed that the degradation of p53 is a consequence of the recruitment and misregulation of ASPP2 by CagA. As a result, CagA-mediated degradation of p53 leads to resistance to apoptosis (Fig. 2d) [96]. Recently, CagA- and ASPP2-interacting domains have been identified. The obtained co-crystal structure revealed that N-terminal subdomain of CagA forms a highly specialized three-helix bundle and that ASPP2 forms an extended helix in this groove of CagA. Consistent with previous reports, this study provides evidence that the direct interaction of CagA and ASPP2 also has an antiapoptotic effect during *H. pylori* infection [97].

P53 is inactivated by mutations in 40 %-50 % of gastric cancers. Individuals infected with CagA-positive *H. pylori* show a higher likelihood of harboring p53 mutations [98, 99]. Activation-induced cytidine deaminase (AID) is a DNA and RNA mutator enzyme. In vitro studies have revealed that CagA-positive *H. pylori* induces aberrant expression of AID in gastric epithelial cells via NF-κB activation and thereby elicits a high mutation frequency in p53 (Fig. 2e) [100]. In addition, AID expression is elevated in *H. pylori*-positive human gastric mucosae and is reduced following *H. pylori* eradication [101]. A recent study in which whole-exome sequencing was performed indicated that p53 mutations accumulate in patients with *H. pylori* infection. In this study, the authors also used AID-transgenic mice to confirm that AID expression plays a critical role in the accumulation of p53 mutations [102]. These studies provide a mutation-dependent mechanism of H. pylori-mediated p53 inactivation.

Similar to p53, (i) Runt-related transcription factor 3 (RUNX3) is also a tumor suppressor, (ii) infection with CagA-positive *H. pylori* is associated with inactivation of RUNX3 in premalignant gastric lesions [103], (iii) CagA inhibits the expression of RUNX3 via the ERK/MAPK signaling pathway [104], (iv) CagA may increase the risk of RUNX3 promoter methylation [105], (v) CagA targets RUNX3 for ubiquitination and proteasome-mediated degradation [106]. In addition, the methylation of tumor suppressor genes is widespread in *H. pylori*-infected models. Cheng et al. used integrative genome-wide scans to identify genes that were concomitantly hypermethylated in mouse and human gastric cancer samples infected with *H. pylori*. They observed that the promoter hypermethylation of the Foxd3 tumor suppressor initiated by *H. pylori* infection affected the prognosis of gastric cancer patients [107]. *H. pylori* infection causes gastric mucosal inflammatory responses, resulting in upregulation of IL-1β and overproduction of nitric oxide (NO). IL-1β and NO play an important role in *H. pylori*-induced methylation [108, 109]. Here, *H. pylori*-induced promoter methylation is observed not only in tumor suppressor genes but also in microRNAs (miRNAs). Silencing of these miRNAs promotes tumorigenesis through activation of their target oncogenes [110]. In general, CagA can decrease the levels of tumor suppressor proteins or inhibit their activity to inactivate of tumor suppressor pathways in a variety of ways.

## Conclusions

Studies in diverse cell lines and animal models indicate that CagA is indispensable for *H. pylori*-induced tumorigenesis of gastric cancer. CagA acts as an initiator that activates multiple host cell signaling pathways via direct or indirect impacts on vital signaling proteins, thereby leading to signaling pathway-dependent oncogene upregulation (Table 1). In addition, CagA also acts as a repressor that inactivates tumor suppressor pathways. As a result, CagA promotes cell proliferation, transdifferentiation and reduces apoptosis, which is beneficial to tumorigenesis. Furthermore, CagA induces cell polarity and morphogenic changes, such as cell motility and scattering (known as the ‘hummingbird phenotype’) and the epithelial-mesenchymal transition, promoting development of gastric cancer. Accordingly, current guidelines strongly recommend treatments aimed at *H. pylori* eradication to prevent gastric cancer. However, few individuals infected by *H. pylori* may develop gastric cancer. Therefore, targeted CagA-positive *H. pylori* eradication may be more suitable for current personalized treatment strategies. A CagA detection kit has been used in clinical practice, but it is regrettable that there are currently no drugs that target CagA-positive *H. pylori*. On the other hand, the *H. pylori*-infected population is susceptible, and these individuals are easily reinfected with *H. pylori* after *H. pylori* eradication. *H. pylori* has coevolved alongside humans to promote persistent colonization of gastric mucosae. Focusing on the pathways implicated in *H. pylori*-induced tumorigenesis may lead to novel therapeutic strategies for gastric cancer prevention.

**Table 1:** CagA-positive H.pylori mediates dysregulation of multiple signaling pathways

Dysregulation of signaling pathway	Molecular mechanism	Proposed function	References
Wnt/β-catenin signaling pathway	Competitive binding E-cadherin	Release of β-catenin from the E-cadherin/β-catenin complex	[22]
	Phosphorylation of LRP6	Activation of Dvl	[34]
	Phosphorylation of Akt	Inactivation of GSK-3β and activation of downstream β-catenin	[37]
	Direct binding GSK-3β	Degradation of GSK-3β and activation of downstream β-catenin	[38]
P13K/Akt signaling pathway	Phosphorylation of EGFR	Activation of the P13K p85 subunit and downstream Akt	[37, 48]
	Interaction with c-met	Activation of P13K/Akt, the downstream β-catenin and NF-κB	[21]
	Interaction with P13K p85	Activation of the P13K p85 subunit and downstream Akt	[20, 50]
NF-κB signaling pathway	Activation of MEK/ERK	Phosphorylation of p65, induced the release of IL-8	[56, 57]
	Autophosphorylation of TAK1 Synergy with phosphorylation of MEKK3	Recruitment and activation of IKK complex	[60]
Shh signaling pathway	Activation of NF-κB	Overexpression of Shh	
JNK signaling pathway	Activation of TNF homolog Eiger or overexpression of Rhol	Upregulation of JNK signaling, induced apoptosis and compensatory proliferation	[68, 69]
JAK/STAT3 signaling pathway	Induced IL-6, IL-10	Phosphorylation of STAT3, nuclear translocation of STAT3	[70–73]
ERK/MAPK signaling pathway	Interaction with SHP2, Grb2 and Crk/Crk-L	Activation of ERK/MAPK signaling, induced cell scattering	[19, 20, 76]

## Abbreviations

*H. pylori*: *Helicobacter pylori*
cag PAI: cag pathogenicity island
VacA: Vacuolating cytotoxin A
CagA: Cytotoxin-associated gene A
T4SS: Type IV secretion system
PS: Phosphatidylserine
ERK: Extracellular signal-regulated kinase
MAPK: Mitogen-activated protein kinase
PI3K/Akt: Phosphatidylinositol 3-kinase/Akt
NF-κB: Nuclear factor-κB
Fzd: Frizzled
LRP: Low-density lipoprotein receptor related protein
Dvl: Disheveled
GSK-3β: Glycogen synthase kinase-3β
APC: Adenomatosis polyposis coli
CK-1: Casein kinase-1
WIF-1: Wnt inhibitory factor-1
sFRPs: Secreted Frizzled related proteins
DKKs: Dickkopf protein family
TCF/LEF: T cell factor/lymphoid enhancer factor
PIP3: Phosphatidylinositol 3, 4, 5-triphosphate
PH: Plekstrin homology
mTOR: Mammalian target of rapamycin
EGFR: Epidermal growth factor receptor
TPMs: Tyrosine phosphorylation motifs
IKK: IκB kinase
TAK1: Transforming growth factor-β-activated kinase 1
FoxO: Forkhead transcription factors of class O
Hh: Hedgehog
Shh: Sonic hedgehog
JNK: c-Jun NH2-terminal kinase
STAT3: Signal transducers and activators of transcription 3
REG: Regenerating islet-derived
DCs: Dendritic cells
BMDCs: Bone marrow-derived dendritic cells
CAFs: Cancer-associated fibroblasts
FAK: Focal adhesion kinase
PKC: Protein kinase C
HDM2: Human double minute 2
ASPP2: Apoptosis-stimulating protein of p53
AID: Activation-induced cytidine deaminase
RUNX3: Runt-related transcription factor 3
NO: Nitric oxide
MiRNAs: microRNAs
